# Management of sexual dysfunction in postmenopausal breast cancer patients taking adjuvant aromatase inhibitor therapy

**DOI:** 10.3747/co.2007.151

**Published:** 2007-12

**Authors:** C. Derzko, S. Elliott, W. Lam

**Affiliations:** * Obstetrics and Gynecology and Reproductive Endocrinology, St. Michael’s Hospital, and University of Toronto, Toronto, Ontario; † BC Center for Sexual Medicine, Vancouver Hospital, and Departments of Psychiatry and Urology, University of British Columbia, Vancouver, British Columbia.; ‡ Burnaby Hospital Regional Cancer Centre, Burnaby, British Columbia

**Keywords:** Aromatase inhibitor therapy, breast cancer, gynecologic side effects, hormone therapy, sexual dysfunction, side effect management, side effect treatment

## Abstract

Treatment with aromatase inhibitors for postmenopausal women with breast cancer has been shown to reduce or obviate invasive procedures such as hysteroscopy or curettage associated with tamoxifen-induced endometrial abnormalities. The side effect of upfront aromatase inhibitors, diminished estrogen synthesis, is similar to that seen with the natural events of aging. The consequences often include vasomotor symptoms (hot flushes) and vaginal dryness and atrophy, which in turn may result in cystitis and vaginitis. Not surprisingly, painful intercourse (dyspareunia) and loss of sexual interest (decreased libido) frequently occur as well. Various interventions, both non-hormonal and hormonal, are currently available to manage these problems. The purpose of the present review is to provide the practitioner with a wide array of management options to assist in treating the sexual consequences of aromatase inhibitors. The suggestions in this review are based on recent literature and on the recommendations set forth both by the North American Menopause Association and in the clinical practice guidelines of the Society of Gynaecologists and Obstetricians of Canada. The complexity of female sexual dysfunction necessitates a biopsychosocial approach to assessment and management alike, with interventions ranging from education and lifestyle changes to sexual counselling, pelvic floor therapies, sexual aids, medications, and dietary supplements—all of which have been reported to have a variable, but often successful, effect on symptom amelioration. Although the use of specific hormone replacement—most commonly local estrogen, and less commonly, systemic estrogen with or without an androgen, progesterone, or the additional of an androgen in an estrogenized woman (or a combination)—may be highly effective, the concern remains that in patients with estrogen-dependent breast cancer, including those receiving anti-estrogenic adjuvant therapies, the use of these hormones may be attended with potential risk. Therefore, non-hormonal alternatives should in all cases be initially tried with the expectation that symptomatic relief can often be achieved.

First-line therapy for urogenital symptoms, notably vaginal dryness and dyspareunia, should be the non-hormonal group of preparations such as moisturizers and precoital vaginal lubricants. In patients with estrogen-dependent breast cancer (notably those receiving anti-estrogenic adjuvant therapies) and severely symptomatic vaginal atrophy that fails to respond to non-hormonal options, menopausal hormone replacement or prescription vaginal estrogen therapy may considered. Systemic estrogen may be associated with risk and thus is best avoided. Judicious use of hormones may be appropriate in the well-informed patient who gives informed consent, but given the potential risk, these agents should be prescribed only after mutual agreement of the patient and her oncologist.

## 1. INTRODUCTION

With more than a million new cases and more than 400,000 deaths worldwide annually, breast cancer is a major cause of morbidity and mortality^1^. The clinical efficacy of adjuvant endocrine therapies in hormone-dependent breast cancer is the key to successful intervention. However, therapeutic standards and guidelines also emphasize that effective patient compliance requires that safety and tolerability be addressed.

Because of their greater efficacy in reducing recurrences as well as their better tolerability profile, the third-generation aromatase inhibitors (ais), including anastrozole (Arimidex: AstraZeneca, Mississauga, ON), letrozole (Femara: Novartis Pharmaceuticals Canada, Dorval, QC), and exemestane (Aromasin: Pfizer Canada, Kirkland, QC), are increasingly being recommended as adjuvant therapy in postmenopausal women with breast cancer. In contrast to a selective estrogen receptor modulator such as tamoxifen, which acts as an estrogen agonist in some tissues (for example, bone or endometrium) and an antagonist in others (for example, breast), ais produce profound suppression of estrogen in all tissues by blocking the cytochrome P450 aromatase complex that converts androgens to estradiol, which underlies the estrogen receptor (er)–positive mammary carcinogenesis^2^–^4^.

The steroidal inhibitor exemestane, at a single oral dose of 25 mg, leads to prolonged reduction in plasma and urinary estrogen levels, with maximal suppression of circulating estrogens occurring 2–3 days after dosing and persisting for 4–5 days^3^. Exemestane inactivates aromatase irreversibly, inhibiting peripheral aromatase by 97%–98%^4^. The nonsteroidal ais—anastrozole, at a dose of 1 mg daily, and letrozole, at a dose of 2.5 mg daily—produce nearly 97%–99% inhibition of estrogen biosynthesis^5^,^6^, suppressing total-body aromatization, plasma estrone, and estradiol levels in postmenopausal women with metastatic breast cancer^5^,^7^. Furthermore, these third-generation ais do not compromise glucocorticoid or mineralo-corticoid production or thyroid function^8^,^9^.

Tamoxifen exerts its estrogen agonist effect by binding to the er on endometrial epithelial and stromal cells, thereby stimulating abnormal cellular proliferation and increasing the risk of polyps, hyperplasia, and endometrial cancer by a multiple of 2–4 times compared with patients not receiving tamoxifen^10^–^18^. Overall, nearly 10% of tamoxifen-treated patients develop endometrial pathology within 5 years^19^. Within 3 months of initiation of tamoxifen therapy, significant increases in endometrial thickness and in uterine volume are reported, including endometrial cysts and polyps and growth of pre-existing fibroids^20^.

The American Society of Clinical Oncology Technology Assessment Panel (2005) supports the use of ais as appropriate initial treatment for women with contraindications to tamoxifen, or for example, in women who develop invasive breast cancer while taking a selective estrogen receptor modulator for breast cancer reduction or bone loss^21^. Clinical studies have clearly demonstrated the safety of steroidal and nonsteroidal ais from a gynecologic viewpoint.

## 2. AIs AND GYNECOLOGIC HEALTH

Data from the four major phase iii trials of ais, including the Arimidex, Tamoxifen, Alone or in Combination study (atac), ma.17, the Breast International Group (big) study 1–98, and the International Exemestane Study, have shown a lower incidence of endometrial cancer and vaginal bleeding or discharge with the ais than with tamoxifen treatment 22,23.

Each of the ais is associated with its own side effect profile. For most women, the ai-induced hypoestrogenic side effects are manageable. Nonetheless, to appropriately address side effects, a careful and comprehensive clinical evaluation needs to be undertaken to determine which of the gynecologic symptoms are attributable to menopause and which are attributable to other causes.

### 2.1 Anastrozole (Arimidex)

Results from the atac trial, investigating upfront treatment with anastrozole as compared with initial tamoxifen therapy in postmenopausal women with early breast cancer, showed a significantly lower incidence of hot flushes, endometrial cancer, and vaginal bleeding and discharge with anastrozole^24^. As well, anastrozole was associated with significantly fewer adverse gynecologic events in four categories^25^:

- Vaginal haemorrhage with no definitive diagnosis: 115 vs. 186, *p* < 0.0001
- Leucorrhea (discharge from the vagina or uterine cavity, or both): 61 vs. 218, *p* < 0.0001)
- Endometrial hyperplasia: 16 vs. 136, *p* < 0.0001
- Endometrial neoplasia: 23 vs. 118, *p* < 0.0001 (Distler D, on behalf of the atac Trialists’ Group. Fewer gynaecological adverse events, gynaecological intervention, endometrial changes and abnormalities with anastrozole than with tamoxifen: findings from the atac trial. Poster presented at the 10th International St. Gallen Oncology Conference; St. Gallen, Switzerland; March 14–17, 2007)

**FIGURE 1:**
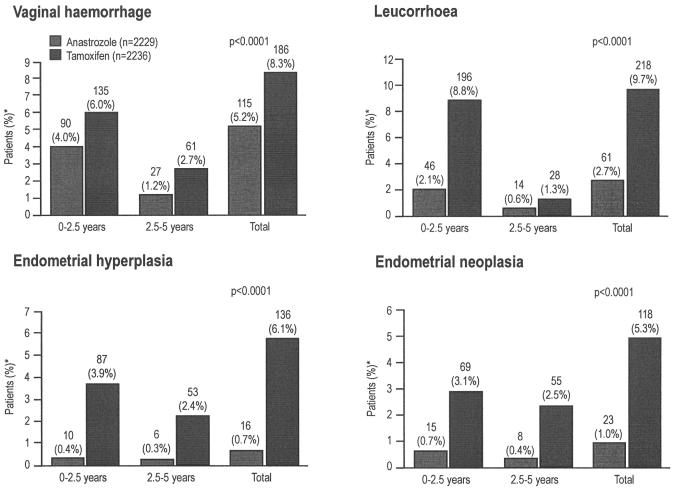
Incidence of specific gynecologic adverse events having a lower recorded incidence with anastrozole use than with tamoxifen use (>3% total difference), by time of occurrence in patients with an intact uterus at baseline in the Arimidex, Tamoxifen, Alone or in Combination main trial (Distler D, on behalf of the atac Trialists’ Group. Fewer gynaecological adverse events, gynaecological intervention, endometrial changes and abnormalities with anastrozole than with tamoxifen: findings from the atac trial. Poster presented at the 10th International St. Gallen Oncology Conference; St. Gallen, Switzerland; March 14–17, 2007). *Patients can have an event more than once, but in different time categories.

Notable also was the fact that, of the patients who experienced gynecologic adverse events, those on anastrozole required fewer diagnostic (21.8% vs. 29.4%) and therapeutic (8.4% vs. 15.4%) interventions than did those on tamoxifen. Furthermore, patients on anastrozole underwent hysterectomy at a quarter of the frequency seen among patients on tamoxifen [1.3% vs. 5.1%, *p* < 0.0001 (Distler D, on behalf of the atac Trialists’ Group. Fewer gynaecological adverse events, gynaecological intervention, endometrial changes and abnormalities with anastrozole than with tamoxifen: findings from the atac trial. Poster presented at the 10th International St. Gallen Oncology Conference; St. Gallen, Switzerland; March 14–17, 2007)]. Most of the gynecologic adverse events and endometrial abnormalities in the atac trial occurred within the first 2.5 years of tamoxifen therapy (Figure 1). The subprotocol analyses also revealed both fewer endometrial abnormalities and fewer medical interventions during the first 2 years with anastrozole therapy than with tamoxifen^26^. This finding suggests that starting postmenopausal patients with early breast cancer on an ai upfront, rather than initiating adjuvant treatment with tamoxifen with the intention of changing to an ai after 2–3 years may be advantageous^25^ (Distler D, on behalf of the atac Trialists’ Group. Fewer gynaecological adverse events, gynaecological intervention, endometrial changes and abnormalities with anastrozole than with tamoxifen: findings from the atac trial. Poster presented at the 10th International St. Gallen Oncology Conference; St. Gallen, Switzerland; March 14–17, 2007).

These results are consistent with findings from an open-label randomized trial that compared, in postmenopausal breast cancer patients, the effects of switching to anastrozole following adjuvant tamoxifen treatment (after more than 12 months, but fewer than 48 months) with the effects of continued use of tamoxifen, histologically confirming tamoxifen-induced endometrial pathology (polyps, hyperplasia, and glandulocystic atrophy)^27^. The difference between groups in recurrent vaginal bleeding [anastrozole: 4 of 83 patients (4.8%); tamoxifen: 9 of 88 patients (10.2%)] was not significant (*p* = 0.18). However, 6 months later, mean endometrial thickness was significantly less in patients who switched to anastrozole than in those who continued tamoxifen treatment (*p* < 0.0001). Also, significantly fewer patients who switched to anastrozole required repeat hysteroscopy or dilatation and curettage as compared with patients who continued on tamoxifen [4 of 83 patients (4.8%) and 29 of 88 patients (33.0%) respectively; *p* < 0.0001)^27^.

**FIGURE 2:**
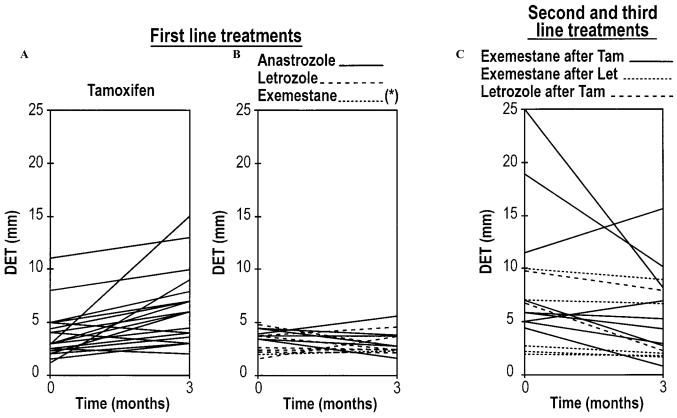
Changes in double endometrial thickness (DET) from baseline to 3 months of treatment with tamoxifen and with aromatase inhibitors^20^.

By inhibiting estrogen synthesis, the third-generation ais reduce endometrial thickness and uterine volume in patients previously treated with tamoxifen (Figure 2)^20^. However, patients receiving anastrozole alone reported more vaginal dryness, painful intercourse (dyspareunia), and loss of sexual interest, but significantly fewer cold sweats and less vaginal discharge^28^,^29^ than did patients on tamoxifen alone.

### 2.2 Letrozole (Femara)

For patients who are intolerant to tamoxifen, switching to letrozole has been shown to reduce the frequency of hot flashes^30^. In the big 1–98 trial, patients on letrozole experienced fewer hot flashes than did those on tamoxifen (33.6% vs. 38.1%)^31^. However, in the ma.17 trial, letrozole was associated with a higher incidence of hot flashes (58%) but with less frequent vaginal bleeding than was placebo (54%)^32^. In subset analyses, women receiving letrozole experienced more bodily pain and menopausal symptoms and a greater compromise in sexual function^33^.

### 2.3 Exemestane (Aromasin)

As compared with tamoxifen, the steroidal aromatase inhibitor exemestane has been shown to result in more frequent vaginal dryness, but less vaginal discharge and bleeding^34^,^35^.

## 3. FEMALE SEXUAL DYSFUNCTION

The gynecologic signs and symptoms associated with diminished estrogen levels, such as urogenital atrophy, vaginitis, dyspareunia, and loss of sexual interest, may affect quality of life to varying degrees in postmenopausal women receiving adjuvant ai therapy.

Female sexual dysfunction (fsd) is multifactorial, involving physiologic, psychologic, social, and emotional components. Delineation into four diagnostic groups is clinically helpful^36^,^37^:

- Hypoactive sexual desire disorder (hsdd)
- Female sexual arousal disorder (fsad)
- Orgasmic disorder
- Sexual pain disorder

Many physiologic, psychologic, interpersonal, and sociocultural factors may contribute to fsd (Table I) 38. These can range from age- or illness-related causes to results of surgical or therapeutic interventions. In addition, sexual motivation and performance can be affected by psychosexual issues. Having breast cancer is itself an additional factor in fsd. Breast cancer treatment may cause early and more severe menopausal symptoms, as well as fear and anxiety, body-image concerns, and sexual dysfunctions arising from cancer treatment–induced urogenital changes.

A variety of female sex hormones, including estrogens, androgens, progesterone, prolactin, oxytocin, and glucocorticosteroids, have been implicated in normal female sexual function. As they interact with neurotransmitters such as serotonin, catecholamines, and dopamine within the central and peripheral nervous system, each of these hormones and their receptors are regulated by co-activator and co-repressor proteins through both endothelium-dependent and -independent mechanisms^39^. Furthermore, steroid hormones can be produced within cells in peripheral target tissues and remain there. Although these hormones are not found within the systemic circulation, they are believed to exert an important local effect—a process called intracrinology^40^. The specific roles of these individual hormones in sexual responsiveness have been difficult to identify clearly.

### 3.1 Clinical Assessment of FSD

Complaints of sexual dissatisfaction or dysfunction in postmenopausal women merit thorough evaluation:

- A sexual history is critical, and self-report questionnaires (see Appendix A) can also be used to assist with the assessment 41–43. General, medical, surgical, psychiatric, psychosexual, and relationship factors should be evaluated.
- Physical examination, including a gynecologic examination, should be done.
- Hormonal evaluation may also be indicated, because levels of a number of relevant hormones may be altered in women on ais 44.

Unquestionably, local (vaginal) factors should be addressed in ai-treated women, as well as in all women with sexual difficulties. If local factors are deemed to be an issue, local treatment with vaginal moisturizers and precoital lubricants should be initiated. If vaginal dryness or lack of elasticity with resulting dyspareunia persist, and if the resulting sexual dysfunction is distressing to the patient, local treatment with vaginal estrogens may be considered. Consideration of systemic estrogen therapies, rarely indicated for vaginal complaints, should be reserved for those women whose severe and distressing systemic symptoms (for example, severe vasomotor disturbances) fail to respond to non-hormonal therapies (for example, clonidine, selective serotonin reuptake inhibitors, selective norepinephrine reuptake inhibitors). Consultation with the patient’s oncologist should precede initiation of hormonal therapy.

#### 3.1.1 The Sexual History

Obtaining a detailed sexual history is not only critical to addressing appropriate sexual concerns, it is also therapeutic in itself. Faced with a life-threatening disease, women with breast cancer may feel that their altered sexuality is not a medically legitimate complaint, or that their health care professionals view sexual changes as irrelevant to other issues or are uncomfortable addressing sexual issues. However, sexual dysfunction is well known to interfere with both quality of life and the recognized need for intimacy during cancer diagnosis and treatment. It is therefore important to include questions that address the possibility of sexual concerns. (For example, “Many women with your diagnosis have sexual concerns. Do you? Many patients may be reluctant to mention it themselves.”) Sexual histories should be taken in the context of current medications and stage of cancer treatment. Cancer treatments and reduced hormone levels may result in lowered mood, fatigue, and loss of sexual interest not only because of the aforementioned stressors, but also because of a reduction in sexually satisfying experiences as a result of dyspareunia or other gynecologic effects of cancer treatment.

History-taking in regard to each sexual concern should include

- delineation of the concern (interest, arousal, orgasm, pain);
- appraisal of precipitating factors and duration of the concern;
- evaluation of whether the sexual concern arises only in some situations or is generalized in its presentation;
- description of the concern (how and when the concern appears and how it plays out in sexual situations);
- assessment of whether the sexual concern affects another aspect of sexual response (that is, low sexual drive may precipitate arousal or orgasmic disorders);
- determination of whether the partner has sexual concerns that may contribute to the woman’s sexual dissatisfaction;
- review of the sexual context within which the woman participates (that is, frequency of sexual activity or attempts, effectiveness of sexual techniques, quality of personal relationship with partner, self and partner expectations); and
- appraisal of previous therapies for the sexual concerns under discussion and the motivation to pursue symptomatic relief.

With any sexual dysfunction, assessing the resulting degree of personal distress while evaluating the patient for physical, psychosocial, organic, and biologic aberrations is important^45^. Screening for depression, chronic illness, or therapy causing adrenal suppression is essential. In regard to the latter, results from a randomized double-blind multicentre trial indicate that ais such as oral letrozole at daily doses of 0.5 mg and 2.5 mg suppressed estrogen levels at both doses without affecting adrenal activity in postmenopausal advanced breast cancer patients progressing after tamoxifen^9^.

Finally, from a medical viewpoint, sexuality encompasses more than genital functioning. Bladder and bowel issues, altered genital sensation, mobility limitations, pain from concomitant medical conditions, presence of a non-cancer-related chronic illness, fatigue, use of medications other than those for cancer, sexual self-image and self-esteem, partnership, motherhood, and parenting issues all affect the sense of sexual self and sexual functioning^46^. It is advisable to ask patients questions about these issues. The next subsection illustrates an approach that can be used.

#### 3.1.2 Physical Examination for FSD

General assessment should include thyroid status; cardiovascular, musculoskeletal, and neurologic parameters; breast examination for further pathology or hyperprolactinemia (nipple discharge); and signs of anemia.

A complete gynaecologic assessment should encompass these possible sources of sexual difficulties^42^,^47^:

- Vulvovaginal estratrophy (introital narrowing, vaginal pallor, dryness, loss of rugosity, friability)
- Abnormal discharge (bubbly, cheesy, foul-smelling)
- Vaginal or perineal lesions (for example, condylomata acuminata, herpes, trauma)
- Size and elasticity of introitus (any atrophy, lesions, scarring, or strictures)
- Discharge or evidence of infection, vulvodynia, and deep tenderness
- Sparse hair of the mons pubis (may suggest low androgen levels)
- Vulvar skin lesions indicating possible infection (Candida, herpes), dermatitis (eczema, psoriasis, allergic reaction), or dermatoses (lichens)
- Atrophy, lesions, or adhesions of the labia majora and minora
- Phimosis and adhesions of the clitoris and female genital surgery (including circumcision)
- Infection or prolapse of the urethra
- Vulvovestibulitis of the vulva and bulbourethral glands (by swab)
- Possible cystocele, rectocele, uterine prolapse, or urinary incontinence (Valsalva manoeuvre)
- Pain or masses (internal bimanual examination) or problems with vaginal tissues and cervix (speculum examination)

Impaired vaginal and rectal muscle tone, pelvic floor hypertonicity, or poor or absent bulbocavernosal reflex may suggest possible sources of sexual difficulty.

Table II lists suggested biochemical investigations^45^. Drugs such as ais that block ovarian function effectively reduce androgen levels. Finding a low serum testosterone level is neither diagnostic nor predictive of sexual dysfunction. Thus, it is generally agreed that serum testosterone determinations should not be used to make a diagnosis. The major role of testosterone determination is in monitoring testosterone therapy to ensure that testosterone levels remain within the acceptable normal range^48^. Although androgens decline with age in all women, no consensus yet exists on normal age-related levels, because large numbers of control and dysfunctional populations alike need to be screened for levels of androgens and their metabolites. Another challenge is the development of a reliable assay specifically for the lower levels of testosterone found in women^49^. The current view is that the total testosterone assay, which measures both freely circulating testosterone and the portion bound to protein, is inaccurate for women with lower ranges of serum testosterone^49^. In the Canadian context, levels of free testosterone (not bound to sex-hormone globulin) and bioavailable testosterone (free testosterone and testosterone loosely bound to albumin) are considered more indicative of cellular or bioactive status than is the total testosterone level. However, the concept of intracrinology mentioned earlier further confuses the picture. The U.S. Endocrine Society has recommended that, until a well-defined clinical syndrome and normative data on total or free testosterone levels across the lifespan have been established, a diagnosis of “androgen deficiency” in women should not be made on the basis of serum testosterone levels^50^.

In summary, there are four significant challenges to the concept of androgen deficiency in women^50^:

- Testosterone assays are inaccurate, and their results are difficult to interpret.
- The correlation between measured low serum testosterone levels and hsdd libido is at best categorized as “fair.”
- Low serum testosterone levels are rarely the cause of hsdd; nonetheless, it remains possible that treatment with testosterone in women with low desire may prove beneficial for responsive sexual drive and may possibly alter orgasmic attainment if endogenous androgen levels are significantly reduced by cancer treatments.
- Aromatization of androgens by adipose tissue is a concern in women with a history of breast cancer, because androgen administration may affect the plasma and cellular concentrations of estrogens^51^.

In the absence of specific guidelines or data regarding the prescription of testosterone without estrogen or the absence of any long-term safety or efficacy data and the inability to unequivocally determine androgen deficiency in women with acquired sexual dysfunction, the sogc recommends that clinicians should exercise caution and seek the patient’s informed consent when initiating testosterone treatment^43^. This partnership approach is particularly important for breast cancer patients, because, notably, concerns still surround the immediate and long-term safety issues related to aromatization of androgens to estrogen for women with breast cancer.

## 4. APPROACH TO MANAGEMENT OF SEXUAL DYSFUNCTIONS

The multifactorial nature of female sexual concerns defies a quick fix; therapeutic interventions should be tailored to address each area of distress (psychologic, interpersonal, sociocultural, and physiologic) and to attend to each affected functional domain (desire, arousal, orgasm, pain).

Available modalities range from education, counselling, and lifestyle interventions to mechanical devices, pelvic floor exercises, and medications^38^. Summaries of therapeutic approaches to the various aspects of sexuality follow. Pharmaceutical interventions are subsequently discussed.

### 4.1 Sexual Disinterest

Women should be advised that an age-related lowered spontaneous (that is, biologically driven urge) sex drive is not abnormal. It is important that breast cancer patients, including those on ai therapy, be made aware of this fact. Although women may choose to be sexual in answer to a biologic sexual urge, they may also be motivated to be sexual for reasons other than sexual drive (generation of emotional closeness, reassurance of a loving relationship despite a diagnosis of cancer or a disfigurement, partner satisfaction, and so on). Although these behaviours are quite normal, it is equally important not to pathologize disinclination to engage in sexual behaviour unless that disinclination is distressing to the woman, because her response may be adaptive rather than dysfunctional^52^.

Various models have been used to describe the female sexual response^53^–^55^. These include the linear model described by Masters and Johnson, whereby genital arousal builds and culminates in orgasm^53^; a single normative (spontaneous urge) responsive pattern driven by sexual desire leading to arousal and orgasm^54^; and a circular model, whereby arousal and responsive drive may not be driven primarily by urge but by other motives, such as intimacy need^55^. In a large community sample of nurses with and without sexual concerns who were asked to endorse one model that best described their sexuality, all models were endorsed equally, demonstrating the heterogeneity of female sexual response; however, the women who endorsed the circular model tended to be women whose sexual experiences were more problematic and unsatisfying (that is, women with lower Female Sexual Function Index scores and those who reported more dissatisfaction with their emotional relationship with their partner)^56^. Thus, a woman’s past sexual experience and present sexual context is important in determining her current sexual life expectations. In assessing sexual concerns, the key elements to discover are what has changed or what is missing (or both), how satisfying current sexual experiences are, and how distressed the woman is about the changes (that is, what the consequences of current circumstances are). Medically, it is important to seek out and address any potentially reversible causes (that is, dyspareunia, depression, hormonal alterations, medication use) and to retain lifestyle and counselling assistance with adaptation to the changes if they persist. Cognitive behavioural therapy and other psychological interventions are often used clinically in the management of low desire, but outcome studies are few to date.

### 4.2 Sexual Arousal and Orgasmic Problems

Arousal problems may stem from inadequate physiologic support to allow for proper vasocongestion, inadequate mental and physical sexual stimulation, or distracting factors such as stress or fear of pain or of bladder incontinence. (See mitigation suggestions later in this subsection.)

Vaginal dryness during sexual activity can be treated symptomatically with commercial lubricants. Various products are available, and most women prefer an odourless, non-irritating preparation. To avoid staining bed sheets or clothing, water-based lubricants should be used with latex condoms. Patients could try thin, water-based lubricants such as Astroglide (Biofilm, Vista, CA, U.S.A.) or KY Liquid (KY, Guelph, ON), or glycerine-free water-based lubricants such as Slippery Stuff (Wal-Med, Puyallup, WA, U.S.A.), Liquid Silk and Maximus Liquid Silk (Bodywise, Cowes, U.K.), Sliquid (Sliquid llc, Dallas, TX, U.S.A.), and Hathor Aphrodesia (Hathor Skin Care, Vancouver, BC), because these products are less likely to cause allergic reactions. The patient—and her partner—should be encouraged to check for new products available in their pharmacy.

Increased intensity and duration of genital (especially clitoral) stimulation may be required for arousal and orgasm. Sometimes couples sexual therapy to instigate better understanding of the woman’s needs may allow for improved partner technique. High intensity vibrators for self or partnered sexual activity have proven successful in allowing some women to obtain more stimulation than can be achieved by intercourse or hand or oral stimulation alone, especially when placed directly on or near the clitoris, or even intravaginally^57^. Several can be discreetly purchased from Web-based companies. The Eros Clitoral Therapy Device (UroMetrics, St. Paul, MN, U.S.A.), a small clitoral vacuum pump used for women with and without fsad to increase sensation, vaginal lubrication, orgasmic attainment, and greater overall sexual satisfaction, has been approved by the U.S. Food and Drug Administration (fda). In select women, it may be beneficial.

Well-controlled randomized trials of psychosocial intervention for fsad in women with cancer are lacking. Recently, however, Brotto and colleagues used a brief, three-session psycho-educational intervention (ped) in 22 women with early-stage gynecologic cancer who had fsad and found a significant positive effect of the ped on sexual desire, arousal, orgasmic satisfaction, sexual distress, depression, and overall wellbeing, and a trend toward significantly improved physiologic genital arousal and perceived genital arousal^58^.

### 4.3 Pelvic Floor Exercises for Sexual Pain Disorders

The pelvic floor is innervated by the limbic system and is highly reactive to emotional stimuli; pelvic floor therapies may therefore be effective in dyspareunia. A six-member committee focusing on women’s sexual pain disorders, part of an international consultation of more than 200 multidisciplinary experts from 60 countries, found that distinguishing vaginismus from dyspareunia may be difficult using clinical tools. However, physical therapists can differentiate vaginismic women from matched controls based on muscle tone and strength differences. The committee recommended a revision to the definitions of vaginismus and dyspareunia, integration of treatment approaches, validation of non-specific treatment effects, controlled studies to test interventions, and sex education to help prevent sexual pain^59^. Learned relaxation of the pelvic floor may assist women who experience pain from hypertonicity, provided that the primary reason for the increased tone is identified. In women without pain, improvement of pelvic floor tone and enhancement of orgasm may be achieved through the practice of pelvic floor exercises, including Kegel exercises.

### 4.4 Bladder and Bowel Issues

Discomfort from urinary tract infections, fear of urine leakage with orgasm, and stress incontinence can interfere with sexuality. Based on clinical studies that showed reduced vaginal pH and increased lacto-bacilli following treatment, the sogc guidelines recommend intravaginal estrogen in postmenopausal women to prevent urinary tract infections associated with aging^60^. The previously noted precautions on hormone therapy should be kept in mind when applying these recommendations to women who have been diagnosed with breast cancer.

Bowel constipation and flatulence or diarrhea secondary to medications, alteration in eating patterns, or radiation treatments will also dissuade sexual activity. Alleviation of bladder and bowel symptoms and anxieties can be helpful sexually.

### 4.5 Mobility Challenges and Sensory Alterations

Women with joint pain may have difficulties with thigh abduction for intercourse or may not be able to withstand pelvic or hip pressure from the weight of their partners. The use of analgesics before activity and the use of cushioning and supports for comfortable sexual positioning can be helpful. Also, genital sensitivity may decrease with neurogenic changes of age and lowered testosterone levels, with arousal requiring longer and more direct genital stimulation. It is important that adequate lubrication be present to reduce friction irritability of the genital tissues.

### 4.6 Management of Concomitant Medical Conditions and Non-genital Menopausal Symptoms

Many medications and drug therapies prescribed to treat medical conditions are associated with sexual dysfunction as a result of associated vascular and psychoneuroendocrine mechanisms. A comprehensive assessment of sexual function before and with current antihypertensive, psychopharmacologic, or other medication-based treatment is therefore mandatory^61^. Because many postmenopausal women may have pre-existing pain or soreness, diabetes, or arthritis, they may find it desirable to maximize pain control with analgesics and to enhance mental and muscular relaxation before engaging in sexual activity. Other suggestions may include taking a warm bath, planning for times of lowest fatigue, emptying the bladder, and taking quiet time to remove stressors before engaging in a sexual activity.

Menopausal symptoms can be managed in the same way in women using ais as in women without breast cancer. However, as noted earlier, because of potential risks, the use of any hormone replacement treatment requires both special consideration and caution. Menopausal symptoms, particularly hot flashes, are reported more often and are considered to be more distressing, severe, and longer in duration in women with breast cancer^62^. Therapeutic options for the management of hot flashes in breast cancer survivors include centrally acting agents (for example, venlafaxine, paroxetine, gabapentin, clonidine). Use of non-pharmacologic and complementary or alternative therapies (soy phytoestrogens, black cohosh, and vitamin E) cannot safely be recommended because of a lack of quality evidence (level i or level ii) of either efficacy or of short or long-term safety data on which to base such recommendations. Furthermore, various reports have been surfacing of inaccurate labelling—active ingredients present despite label claims to the contrary—in many such store-bought products. These “natural” products are not under the same regulatory governmental control as are pharmaceuticals^62^.

### 4.7 Sexual Self-esteem and Self-image

Breast surgery, depending on the type and extent, can affect body image and self-esteem, and the issues of body image and sexuality after breast cancer may become increasingly important with time elapsed since surgery^63^. Mastectomy patients have a significantly poorer body image and lowered scores for female role and sexual function as compared with patients who undergo breast-conservative surgery^64^. The vulnerability of both body image and sexuality is greater in patients with advanced breast cancer and in those who need adjuvant treatment, have lymphedema, are sedentary, have poor family and social supports, or are single or in an unstable relationship. Sexual or psychological counselling, or both, are recommended for breast cancer survivors and their partners so as to improve the quality of intimacy and body image^63^.

Not to be forgotten is the physical discomfort and pain arising from mastectomy or breast surgery itself. When felt during intercourse or love-making, this symptom may be upsetting to both the woman with breast cancer and her partner. If the incision or muscles are tender, the patient can minimize the pressure on the chest area by lying on the unaffected side. This position provides the patient with more control over her movements, reducing irritation to the incision. If the partner is on top, the patient can be advised to protect the affected area by putting a hand under her chin, with her arm against her chest. If she feels any pain, she should stop and let her partner know why she is stopping. If the couple have discussed this problem in advance and have clarified that the cancer patient will speak up when she notices any pain, then both will feel more relaxed and less inhibited in exploring and experimenting. Taking a rest or changing position may also help the cancer patient to relax, which may in itself reduce pain. Such a pause also provides an opportunity to apply extra lubrication. With communication and cooperation, the patient and her partner can work together to find positions and activities that give the most pleasure^65^.

### 4.8 Relationship Concerns

Sexual life can be altered significantly with the changed relationship dynamics that may accompany diagnosis and treatment of cancer. Psychosexual therapy for the patient alone or with her partner may ameliorate specific psychologic or interpersonal factors such as relationship distress, extended periods of sexual abstinence, and dysfunctional communication patterns—possibly enhancing sexuality^66^.

## 5. PHARMACOLOGIC THERAPIES FOR FSD

The principles for managing symptoms in women with breast cancer follow the strategies seen in the care of postmenopausal patients with sexual health problems^66^. Multidisciplinary interventions must be considered as needed, and modification of reversible causes includes sex therapy, lubricants, altering medications, modifications in lifestyle, and physical therapy for pelvic floor disorders. At that point, first-line therapies should be administered upon diagnosis, and after needs, expectations, risks, benefits, and costs have been clarified. More invasive second-line therapies are initiated only with failures or insufficient response^66^.

### 5.1 HSDD

**FIGURE 3:**
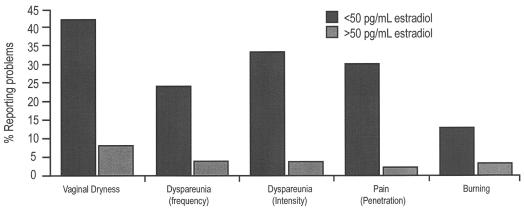
Association between lower estrogen levels and increased prevalence of sexual problems^67^,^68^.

Data suggest that estrogen-deficient postmenopausal women experience an increased incidence of sexual dysfunction and that estrogen or estrogen–progestin therapy (et/ept) may improve or correct the problem (Figure 3)^67^,^68^.

Although the benefits and risks of hormone therapy in 50- to 80-year-old “normal/healthy” women (that is, without breast cancer) continue to be debated, the Women’s Health Initiative^69^ provided no information about the risks of et/ept use in menopausal women with breast cancer. However, two independent randomized controlled trials begun in 1997 studied the question of hormone replacement therapy (hrt) following a diagnosis of breast cancer, and they reached two diametrically opposed conclusions. The habits (Hormonal Replacement Therapy—Is It Safe?) trial was stopped after a median of just 2.1 years because of a statistically significantly higher recurrence of breast cancer in the treated group [relative risk (rr): 3.3; 95% confidence interval (ci): 1.5 to 7.4]^70^. However, the Stockholm Randomized Trial found no significant increase in breast cancer in the treated group at a median of 4.1 years (rr: 0.82; 95% ci: 0.35 to 1.9) 71.

Low-dose, local vaginal estrogen therapy [Premarin cream (Wyeth Pharmaceuticals, Philadelphia, PA, U.S.A.), Vagifem tablets (Novo Nordisk, Princeton, NJ, U.S.A.), Estring (Pfizer Canada), or if available, the more poorly absorbed estriol vaginal cream] is not uncommonly prescribed and may be considered (with the permission of the oncologist) for highly symptomatic er-positive early breast cancer patients who are unresponsive to non-hormonal therapy. However, a decision to use systemic hormone therapy must be undertaken with considerable caution.

Given the uncertainty of the reason for the discordant conclusions of the habits and Stockholm trials, despite the efficacy and numerous benefits of et/ept demonstrated in the early, otherwise healthy, menopausal woman, a decision to use systemic menopausal hormone therapy in a woman with early breast cancer should be made only after careful consideration of the alternatives and consultation with the entire treatment team—including the patient.

In the absence of a biochemical measure that clearly identifies who can be treated, the Endocrine Aspects of Female Sexual Dysfunction Committee suggests that exogenous testosterone should be considered only after other causes of hsdd such as depression, relationship problems, and ill health have been excluded. The Committee recommends comprehensive risk–benefit analysis and informed consent of patients before therapeutic management of hsdd with hormonal therapies is contemplated (summary in Table III)^45^.

A 2005 position statement from the North American Menopause Society (nams) indicates that postmenopausal women may be candidates for testosterone therapy if they present with symptoms of decreased sexual desire associated with personal distress and if they have no other identifiable cause for their sexual concerns^48^. Testosterone therapy is not recommended in the absence of concomitant estrogen therapy, because data on the safety and efficacy of testosterone therapy in women not using concomitant estrogen are lacking. Another nams recommendation is that postmenopausal women undergo laboratory testing for testosterone levels only to monitor for supraphysiologic levels before and during therapy^48^. As noted earlier, any hormonal therapy in the patient treated for breast cancer must be undertaken only with extreme caution.

### 5.2 Androgen Therapies and Breast Cancer

A net decline in testosterone levels occurs following natural menopause. In the premenopausal female, 50% of the circulatory levels of testosterone represent equal contributions from the ovaries and the adrenal glands, but with increasing age, the contributions of the adrenal glands, the peripheral tissue, and the ovaries to dehydroepiandrosterone (dhea) sulphate remain about the same, while the contribution of the ovaries to the testosterone pool increases significantly. Nevertheless, overall levels of androgen decrease because of the precipitous decline in production of testosterone pro-hormones from the adrenal gland—to the extent that the ovaries cannot correct the deficit^72^. The symptoms of low androgen in women are reported to be similar to those in men: a decrease in libido, energy, or sense of wellbeing, and decreased lubrication and arousability even in the presence of estrogens^73^. In the absence of estrogen, and after aromatase conversion, androgens elicit an er-mediated stimulation.

Few studies exist to confirm the benefit of intervention with androgens in breast cancer patients or in postmenopausal women at increased risk for the disease. In one study, during a mean follow-up of 5.8 years, no increase in breast cancer was seen in a group of postmenopausal women when testosterone was added to hrt^74^. A prospective, double-blind study investigated the use of a transdermal nicotine patch (300 μg daily) in postmenopausal women, together with continuous combined estradiol 2 mg and norethisterone acetate 1 mg and randomized additional treatment with either a testosterone or a placebo patch in equal numbers^75^. The findings indicated that the addition of testosterone to a common estrogen–progestogen regimen inhibits the stimulatory effects of hormones on breast cell proliferation^76^.

In a randomized placebo-controlled study investigating dhea 50 mg daily over a period of 12 months, a significant increase in sexual satisfaction and a possible increase in sexual interest and activity was demonstrated in older (ages 70–79 years), but not younger (ages 60–69 years) women^77^. In the absence of any long-term studies with dhea, a risk of ultimate aromatisation to estrogen may have gone undetected, especially in patients already receiving ais for breast cancer. However, one author suggests that “the fear that testosterone [will] be aromatised to estrogen is more paranoia than reality” considering the low levels of conversion to estrone and estradiol that make any risk of breast cancer extremely unlikely^73^. Furthermore, testosterone has been used off-label, without serious safety problems, in postmenopausal women for decades to the benefit of properly selected subjects. Nonetheless, inconclusive results and significant methodologic limitations pertaining to the use of testosterone leave the evidence with regard to breast cancer risk uncertain^78^, and therefore testosterone is not currently recommended for women with breast cancer. Discussion is required before treating this patient population with testosterone therapy.

A recent randomized placebo-controlled phase iii crossover clinical trial randomly assigned 150 partnered postmenopausal women with a history of cancer who were reporting a decrease in sexual desire to a 10-mg equivalent testosterone dose in a cream base [Vanicream (PSI, Rochester, MN, U.S.A.), 2% testosterone] or placebo for 4 weeks each. It was demonstrated that women who were on active testosterone cream had higher serum levels of bioavailable testosterone than did the women on placebo, but the average intra-patient libido change from baseline to weeks 4 and 8 was similar in both arms^79^. No adverse effects on estrogen levels or liver function were noted in this short-term study. The authors suggested that the increased testosterone level did not translate into improved libido possibly because women in the study were estrogen-depleted. In addition to the possible significance of the placebo effect in this study, the study’s null results highlight the complex relationship of biologic, emotional, and cognitive inputs to the perception of desire^80^ and still provide no information about long-term safety of androgen use in women. The fda has already requested more long-term safety data on the testosterone patch Intrinsa (Procter & Gamble Pharmaceuticals, Egham, U.K.) for medical treatment of fsd in menopausal women.

Although naturally postmenopausal women with symptoms of testosterone deficiency despite conventional hormone therapy may benefit from androgen treatment, studies have yet to be done to see this benefit in cancer survivors. Any long-term testosterone therapy requires close monitoring for skin and hair problems, including seborrhea, acne, hirsutism, and androgenic alopecia, and for voice changes. Monitoring for biochemical changes and for free and bioavailable testosterone levels and sex-hormone binding globulin is desirable^45^.

### 5.3 Phosphodiesterase Inhibitors for FSAD

Phosphodiesterase V inhibitors (pde5is) work through the nitric oxide–cyclic guanosine monophosphate pathway to relax genital cavernosal smooth muscle in erectile tissues in both men and women following sexual arousal. The pde5is currently used for the treatment of erectile dysfunction in men include sildenafil (Viagra: Pfizer Canada), vardenafil (Levitra: Bayer, Toronto, ON), and tadalafil (Cialis: Eli Lilly and Company, Toronto, ON). For aroused women, pde5is induced tumescence of the smaller erectile structures, which may or may not make a difference to subjective sexual response. Sildenafil (50 mg, adjustable to 100 mg or 25 mg) has been shown to increase vaginal lubrication, genital sensation, ability to achieve orgasm, and overall satisfaction in a 12-week double-blind placebo-controlled study in 202 postmenopausal women with fsad without concomitant hsdd or contributory emotional, relationship, or historical abuse issues^81^. In another study using a modelling and simulation framework based on telephone sexual activity diary data, a dose-dependent effect was observed with sildenafil cit-rate in patients with fsad.^82^ However, in a large, multicentre, placebo-controlled trial examining the efficacy and safety of sildenafil 10–100 mg taken 1 hour before sexual activity in two groups of women (estrogen-replete, *n* = 577; estrogen-deficient, *n* = 204) with fsad, no increase in sexual arousal was noted at any treatment dose. In addition, side effects included headache, flushing, rhinitis, nausea, visual disturbance, and dyspepsia^83^.

Large, long-term clinical trials to firmly establish the efficacy and safety of these drugs in women have not yet been carried out as they have been in men. But a randomized controlled trial using the technique of vaginal photoplethysmography to study genital vasocongestion demonstrated reduced latency to orgasm in a subgroup of postmenopausal women with acquired genital fsad who were taking sildenafil.^84^ There are also studies showing that sildenafil can reverse antidepressant-induced fsad 85. However, sildenafil is contraindicated for women with recent myocardial infarction or stroke, active coronary ischemia, or episodes of heart failure, and in those receiving nitrates^86^.

Topical prostaglandin E1 (alprostadil) works differently than pde5is do, directly relaxing erectile smooth muscle through the cyclic adenosine monophosphate system and therefore not requiring nitric oxide release from the nerve endings (that is, sexual arousal) to work. In men, prostaglandin E1 is highly effective in intracavernosal penile injection therapy, but less successful when used as an intraurethral suppository or as a penile topical application for erectile dysfunction. Preliminary studies in postmenopausal women with fsad indicate that, as compared with placebo, topical application of alprostadil to the genitalia in doses of 400 μg produced reports of significantly greater physical and emotional arousal and sexual satisfaction^87^; however, these results have been inconsistent and not reproducible in other trials^88^. The results of ongoing clinical studies are needed to further define the role of topical alprostadil in the treatment of fsad.

### 5.4 Bupropion for Sexual Desire, Arousal, Orgasm, and Satisfaction

Sustained-release bupropion (Wellbutrin SR: GlaxoSmithKline, Mississauga, ON) 300 mg daily used for 4 weeks has been shown to increase sexual desire and frequency of sexual activity in patients with antidepressant-induced sexual dysfunction, as measured by the Changes in Sexual Functioning Questionnaire^89^. However, in non-depressed premenopausal women, bupropion significantly increased sexual arousal, orgasm completion, and sexual satisfaction, but not desire^90^. In depressed women, extra-long-release bupropion (Wellbutrin XL) 300 mg daily was found not to change sex functioning scores and had the same antidepressant effect as venlafaxine XR, but without the adverse effects on sexual function of the latter medication^91^. Bupropion can also be added to an antidepressant regimen to attempt to ameliorate some induced side effects. Further studies are needed in view of the risk of seizures, especially in women with eating disorders.

### 5.5 Alternative Therapies

A number of dietary supplements have been investigated for the treatment of fsd. Nutritional supplements containing ginseng, ginkgo, l-arginine, damiana, vitamins, and minerals have also been reported to improve sexual desire, vaginal dryness, clitoral sensation, and frequency of sexual activity in normal female volunteers^92^, but not in women with cancer or receiving ai therapy.

Meanwhile, researchers are exploring the efficacy and safety of the dopaminergic agonist apomorphine (sublingual, intranasal) and of melanocortin-stimulating hormone (intranasal) in the treatment of hsdd. For fsad, studies are underway into selected vascular smooth muscle relaxants, including nitric oxide pathway agents such as arginine and the prostaglandin E1 topical cream described earlier^93^,^94^.

## 6. GYNECOLOGIC MANAGEMENT OF SEXUAL PAIN FROM ATROPHIC VAGINITIS AND VAGINAL ATROPHY

Approximately 40% of postmenopausal women have symptoms of atrophic vaginitis, and yet fewer than 25% of them seek medical help. Although the condition is attributable to estrogen deficiency, anti-estrogenic medications such as tamoxifen (and possibly ais) or medical or surgical conditions that result in decreased levels of estrogen can exacerbate the signs of decreased vaginal lubrication, followed by other vaginal and urinary symptoms aside from concomitant infection (Table IV)^95^.

As the vaginal mucosa becomes thinner and drier because of declining estradiol levels (from 120 ng/L at perimenopause to about 18 ng/L after menopause), vaginal discomfort, dryness, burning, itching, and dyspareunia may occur and usually progress with time. Inflammation of the vaginal epithelium may contribute to urinary symptoms such as increased frequency, urgency, dysuria, incontinence, and recurrent infections in addition to pelvic laxity and stress incontinence. In addition, changes in vaginal pH and vaginal flora may predispose postmenopausal women to urinary tract infection and *Candida* outbreaks^96^.

### 6.1 Evaluation for Infection in Vaginal Atrophy

Vulvar itching, dyspareunia, vulvar and cervical erythema, vaginal inflammation (with or without cervical inflammation), and often a thick (curdled), white, non-malodorous vaginal secretion are typical of candidiasis. Bacterial vaginosis is characterized by a creamy or yellow secretion and a fishy odour, especially when KOH is added to a hanging drop specimen. *Trichomonas vaginalis* is associated with cervical lesions, friability, microhemorrhagic zones, and frothy, greenish, foul-smelling vaginal secretions^97^.

A definitive diagnosis of vaginal infection (most commonly *Candida albicans,* bacterial vaginosis, or *T. vaginalis*) is made in only about one third of post-menopausal women who present with symptoms of vaginitis. In most of these patients, the symptoms are the result of estrogen deficiency or nonaerobic bacterial infections, local irritants, allergens, or dermatologic conditions^98^.

To increase the likelihood of diagnosing vaginal or urogenital atrophy, physicians should routinely inquire about symptoms such as vaginal irritation or dryness, decreased lubrication with coitus, or recurrent bladder infections. In addition, postmenopausal women should be encouraged to report these symptoms if they do occur, because all are readily correctable, but in the absence of treatment they have the potential to adversely affect quality of life.

In postmenopausal women, the underlying risk factors for chronic or recurrent vulvovaginal candidiasis include immunosuppression caused by medication or disease, hrt, and uncontrolled diabetes mellitus^99^. The physiologic effects of chronic stress (such as diagnosis and treatment of breast cancer) inhibit cellular immune responses that are relevant to cancer prognosis^100^. In fact, physical wellbeing, mood, and coping effort may affect, and in turn be affected by, markers of activation of the cellular immune system, as observed at 3 and 6 months of adjuvant therapy in the International Breast Cancer Study Group adjuvant clinical trials^101^.

Postmenopausal patients at risk for recurrent candidiasis should be evaluated and treated the same way as younger women are^99^. In addition to being estrogen-deprived, postmenopausal women with recurrent vulvovaginal candidiasis harbour more aggressive and resistant fungi^102^.

In the diagnosis of vaginal candidiasis, the following symptoms are clinically helpful:

- Vaginal discharge (quantity, color, consistency)—A “cheesy” discharge is quite symptomatic of candidiasis, and a thick, curdy, or flocculent white discharge is strongly predictive of candidiasis; a watery discharge makes candidiasis less probable.
- Itching—Between 70% and 90% of patients complain of itching.
- Inflammation (redness, pain or burning, swelling)—Vulvar or vaginal edema, erythema, fissures, or excoriations are associated with candidiasis, although they can also occur in trichomoniasis.
- Absence of odour (fishy or foul)—A lack of odour indicates an increased likelihood of candidiasis; the presence of an odour perceived by the patient reduces the likelihood of candidiasis.
- Patient’s self-diagnosis—“Another yeast infection” is most probably candidiasis. However, if the condition fails to resolve with over-the-counter treatment, or if it recurs, the patient needs to be examined, and cultures taken.
- Urinary tract symptoms—Women with candidiasis may suffer from “external” dysuria^103^.

In brief, candidiasis is associated with vulvar pruritus, a cheesy discharge, and redness, and it is often identified through self-diagnosis. Bacterial vaginosis is associated with increased discharge and a complaint of odour. However, microscopic findings are not entirely diagnostic, and primary care clinicians need to be skilled at screening for vaginal candidiasis, bacterial vaginosis, and trichomoniasis in postmenopausal women presenting with vulvovaginal complaints^103^.

### 6.2 Clinical Examination for Vaginal Atrophy and Vaginitis

Gynecologic examination aided by an appropriately sized speculum should investigate vulvar skin close to the vagina for signs of dystrophy or other lesions, including premalignant and malignant disease^96^. The estrogen-sensitive labia majora and minora should also be examined. Urethral caruncle, prolapse (cystocele, rectocele, enterocele, and uterine prolapse), the cervix, and pelvic masses should be noted.

### 6.3 Diagnosis of Vaginal Atrophy and Vaginitis

To confirm urogenital estratrophy vaginitis, pelvic examination of the vulva and vagina should show signs of dryness, pallor, redness (if inflamed), and thinning of tissue^95^,^96^. Pale, smooth, shiny, and dry vaginal epithelium suggests atrophy.

Urogenital status can be evaluated using the Vaginal Health Index, which assigns a score to a number of parameters that are used to assess the vaginal epithelium. These include colour, rugosity, moisture and secretion, elasticity, friability to touch, and pH (which ideally should be less than 5). A lower index is indicative of vaginal atrophy of greater severity^96^. Inflammatory signs include patch erythema, petechiae, increased vascularity, friability, and bleeding and discharge, which indicate vaginitis. Vaginal cytology (especially samples taken from the upper third of the vagina) for maturation index that shows increased parabasal and intermediate cells and decreased superficial cells is indicative of lowered estrogen status. The normal acidic vaginal pH of 3.5–4.5 in healthy mature females becomes alkaline after menopause (assessed with a pH indicator strip inserted into the vagina) and predisposes women to recurrent vaginitis and urinary tract infections. However, candidiasis can occur even at normal vaginal pH (≤4.5 or ≤4.9)^103^.

When post-coital bleeding occurs in menopausal women, an endometrial biopsy becomes necessary to rule out endometrial pathology, including cancer. A Pap smear can confirm cervical health. Cervical bleeding may be the result of estratrophy. The finding of a friable cervix mandates exclusion of cervicitis, particularly *Chlamydia*, because cervicitis is a more likely cause of post-coital bleeding than is endometrial carcinoma. Furthermore, bleeding from coital “trauma” in an atrophic, stenotic introitus or vagina suggests that endometrial carcinoma is unlikely. Transvaginal ultrasound assessment of the uterus and particularly of the endometrium is very helpful, both in atrophic and in well estrogenized tissues. If endometrial ultrasound shows a thickened endometrium, biopsy is essential to rule out endometrial pathology, be it hyperplasia, carcinoma, or an endometrial polyp. A thin endometrium (usually defined as an endometrial stripe of 4 mm or less) measured on transvaginal ultrasonography suggests an atrophic or non-stimulated endometrium. Infection with *Trichomonas, Candida,* or bacterial vaginitis may be a cause of post-coital bleeding. Table V provides a differential diagnosis of atrophic vaginitis^95^.

### 6.4 Management Options

The guidelines approved by the sogc recommend initiation of safe and proven therapies for clinical management as indicated by basic pelvic examination, examination of the vulva, and laboratory tests (Table VI)^60^.

#### 6.4.1 Vaginal Atrophy

Options for the management of vaginal atrophy depend on the specific clinical symptoms. Treatment options range from lifestyle modifications to non-hormonal and hormonal interventions.

##### Lifestyle:

The sogc guidelines 60 encourage regular vaginal coital activity to increase blood circulation to the pelvic organs and avoidance of products that pose a risk of contact dermatitis of the vulva. Contact dermatitis of the vulva may be caused by irritants such as perfumed or dyed toilet tissue; tight-fitting garments, underwear, or bathing suits; soaps, detergents, or fabric softeners; talcum powder; hygiene sprays; deodorant pads; spermicidal foams, creams, or jellies; rubber products, including diaphragms or condoms; poison ivy or similar plants; and talcs 104. The sogc has emphasized the lack of evidence to support any beneficial effects of dietary estrogens or supplements such as *dong quai.* However, the sogc does encourage consumption of pure cranberry and lingonberry juice concentrates to avoid urinary tract infections^60^,^105^. Smoking is associated with decreased estrogen levels and consequent vaginal estratrophy—yet another reason to encourage smoking cessation^60^.

##### Vaginal Moisturizer:

A non-hormonal moisturizing gel containing purified water, glycerine, mineral oil, polycarbophil, carbopol 974P, hydrogenated palm oil glyceride, and sorbic acid (Replens: Wellspring Pharmaceutical Corporation, Bradenton, FL, U.S.A.)—the only vaginal moisturizer available in Canada— used three times weekly has proven efficacy in increasing vaginal moisture and vaginal fluid and in decreasing vaginal itching, irritation, and dyspareunia^60^,^106^.

##### Lubricants for Coital Comfort:

Several lubricants that can be used to reduce immediate irritation during coital activity are available to women in Canada, but no evidence currently exists that these products have any long-term therapeutic effect^60^. Water-soluble vaginal lubricants that relieve vaginal dryness and moisten tissue can substitute for estrogen^96^, but most water-based lubricants contain glycerine, and patients prone to yeast infection should avoid them.

##### Lubricants for Vaginal Dryness:

Orally administered or locally applied vitamin E in daily doses of 100–600 IU has been found to increase vaginal lubrication and to relieve the dryness and irritation that accompany atrophic and other forms of vaginitis. KY Jelly (KY) with vitamin E is another possibility. Vitamin D and analogs used in postmenopausal osteoporosis are also involved in growth and differentiation of stratified squamous epithelium of the vagina. Homeopathic and natural health supplements of bryonia, belladonna, lycopodium have been used, although these remedies have failed in randomized trials of safety and efficacy.

##### Hormonal Therapies for Urogenital Atrophy, Vaginitis, and Dyspareunia:

In general, non-hormonal treatments are the first-line recommendation in breast cancer patients^107^,^108^. However, expert recommendations reviewed and revised by the Breast Disease Committee of sogc concluded that hrt after treatment for breast cancer has no adverse impact on recurrence and mortality, and therefore hrt is an option in postmenopausal women with previously treated breast cancer. Careful evaluation of the indication for hrt and limiting the duration of postmenopausal hrt to the shortest duration possible were recommended^37^. However, the findings from the habits and Stockholm trials raise additional questions and emphasize the need for careful consideration, review, and collaborative discussion of all the risks and benefits in each individual case. Some evidence also suggests that the small risk of ovarian cancer and endometrial cancer observed with hrt is evident only with therapies that use estrogen alone^109^.

Because minimal systemic absorption occurs with use of the recommended doses, the opinion of the sogc is that women with a history of breast cancer may still use local intravaginal estrogen preparations in the recommended doses for the treatment of symptoms of urogenital atrophy^110^. Tested topical estrogen replacement therapies available in Canada include a conjugated equine estrogen (cee) cream, vaginal estradiol tablets, and an estradiol-containing Silastic vaginal ring.

Available systemic estrogen therapies (oral and transdermal patches, and gels) reduce vasomotor symptoms (hot flashes) and sleep disturbance. They also estrogenize urogenital tissues, although, not uncommonly, additional local treatment (vaginal tablets, creams, rings) is needed. The Canadian Consensus Conference on Menopause and 2006 Updated sogc guidelines and recommendations^43^ indicate that local estrogen can ameliorate dyspareunia associated with vulvovaginal atrophy. Low doses may suffice. For example, half an applicator of cee cream (Premarin) intravaginally once or twice weekly, or one intravaginal estradiol tablet (Vagifem) twice weekly is usually adequate. On occasion, a small amount of cee cream (less than dime size) applied to the peri-introital and peri-urethral area once weekly, or even less frequently, may provide symptomatic relief with minimal dosing.

An alternative treatment proposed in the sogc guidelines is use of a sustained-release intravaginal ring that slowly and constantly releases 5–10 μg of estradiol daily from its estradiol-loaded core over a 3-month period. Use of the Estring (Pfizer Canada) effectively relieves symptoms of atrophic vaginitis and restores normal vaginal pH and cytology, maintains serum estradiol levels in the menopausal range, and does not induce endometrial proliferation^111^.

Despite the efficacy of vaginal estrogen preparations in treating vaginal atrophy, a small study of 7 postmenopausal women on ais who were using Vagifem vaginal estradiol tablets noted an increase in serum estradiol levels (>19 pmol/L over low baseline postmenopausal estradiol levels) that it was feared might reverse the activity of the ais being used (Table VII) 112. Despite the very small size of this study, physicians are cautioned, in the absence of other reassuring data, to avoid this treatment combination and to recommend non-hormonal preparations instead.

Although good data from Sarrel^68^ and others suggest that estrogen therapy (possibly including vaginal estrogen treatment) improves local genital sensitivity and sexual response, thereby potentially improving sexual interest and motivation^44^, others disagree. Safety is the overriding consideration. In the opinion of the sogc, as reported in their clinical practice guidelines^107^, the presence of estrogens at concentrations considerably higher than those attained with current hrt preparations does not negatively influence the efficacy of breast cancer management, as has already been demonstrated by other studies^113^–^115^. Data from ongoing prospective randomized clinical trials should offer definitive conclusions.

In brief, if appropriate, replacement of missing hormones or enhancement of depleted levels in breast cancer patients can add to quality of life and should be considered in particular for the treatment of vaginal dryness.

## 7. CONCLUSIONS

The gynecologic signs and symptoms associated with diminished estrogen levels can affect, to varying degrees, the quality of life for postmenopausal women receiving adjuvant ai therapy. In addition to the direct sexual side effects related to diminished estrogen, initiation of treatment for breast cancer often results not only in early and more severe menopausal symptoms, but also in fear and anxiety, body image concerns, and sexual dysfunctions related to altered pelvic health. Because of the complexity of fsd, a biopsychosocial approach to assessment and management is necessary. A detailed sexual history addressing specific sexual concerns is a therapeutic intervention in itself. Besides genital functioning, issues affecting sexual health may encompass bladder and bowel difficulties, mobility limitations, pain, fatigue, and issues with self-image and self-esteem.

The ais offer numerous advantages over tamoxifen for the treatment of early breast cancer in post-menopausal women: fewer gynecologic adverse events, fewer hot flashes, fewer endometrial abnormalities (including fewer polyps, less hyperplasia, and a lower incidence of endometrial cancer), and overall, fewer diagnoses requiring a hysterectomy. For the woman with breast cancer, avoiding the invasive and uncomfortable diagnostic and therapeutic interventions associated with uterine abnormalities can have a tremendous impact on quality of life. Clinical evaluation of ai therapy–associated signs and symptoms of urogenital atrophy, vaginitis, dyspareunia, and loss of sexual interest demonstrates several similarities with natural age- and menopause-related gynecologic events associated with diminished estrogen levels. Management of these events through a combination of lifestyle modification, counselling, and hormonal and non-hormonal interventions can therefore improve quality of life significantly for patients.

Despite the lack of a pharmacologic “gold standard” for the treatment of female sexual concerns, therapeutic regimens can be tailored to effectively address each area of distress—psychologic, interpersonal, sociocultural, and physiologic—in the affected functional domains of desire, arousal, and orgasm.

Hormone replacement therapies such as the intravaginal ring with a sustained-release estradiol-loaded core (Estring) recommended by sogc have proven effective in improving patient compliance for symptomatic relief of atrophic vaginitis and in restoring normal vaginal pH and cytology without side effects of endometrial proliferation or a significant rise in systemic estradiol levels. Recommended alternatives are estradiol tablets (Vagifem) and low doses of cee cream (Premarin). Preliminary data suggest that androgens alone or the addition of testosterone to a common estrogen–progestogen regimen may inhibit the stimulatory effects of estrogens and progestins on breast cell proliferation and may, in fact, lead to apoptosis of cancer cells. However, these results are preliminary; long-term safety data are lacking. Furthermore, few studies have looked at the relationship between testosterone therapy and breast cancer risk in postmenopausal women, and thus no definitive answers are available to guide patient management 76.

In view of recent findings raising concerns over elevated circulating estradiol levels in breast cancer patients on ai therapy who are using transvaginal estrogenic preparations, non-hormonal therapies including regular application of vaginal moisturizers and lubricants are recommended and certainly should be first-line therapy. In addition, pelvic therapy for pelvic tone awareness and pelvic floor exercises (for example, Kegel exercises) and lifestyle modification are preferred and should be considered early.

The sexual context within which the patient exists is the key factor that needs to be assessed before any medical intervention is added. Concerns that interfere with sexuality (including bladder and bowel issues, motor and sensory changes, and management of menopausal symptoms in general), other chronic medical conditions, and sexual self-esteem and partner relationships are all importance parts of the patient’s sexual context, and any effective intervention must account for all of them. Psychological intervention and sex counselling are important adjuncts in the comprehensive management of these patients, and interventions should be considered and provided as needed.

## Figures and Tables

**TABLE I:** Factors underlying female sexual dysfunction^38^

Physiologic factors Aging and menopause Normative and gradual decline in desire; decreased genital perfusion, engorgement, and vaginal lubrication, touch perception, and vibratory sensation; decreased muscle tension in the pelvic floor, and decreased uterine contractions during orgasm Endocrine changes Low estrogen levels leading to vaginal dryness, pain during vaginal penetration, and dyspareunia; low androgen levels linked to decreased sexual desire, genital sensation, and genital response Sickness, injury, or disability Neurovascular injury related to (for example) cardiovascular events, arthritis, diabetes mellitus, and pain; limited mobility related to other medical conditions Surgical therapies Surgical menopause, oophorectomy, hysterectomy; postsurgical dyspareunia or orgasmic dysfunction, or damage to pelvic nerves during presurgical procedures Prescription medications Antidepressants (especially selective serotonin reuptake inhibitors and dopamine receptor blockers); central nervous system depressants and estrogen, androgen, or cholinergic antagonists; antihypertensive medications (centrally acting sympatholytic agents, beta-blockers, diuretics) Psychologic factors Defective physical or mental status History of physical or sexual abuse Poor self esteem or self-image Unrealistic goals (long-term relationship and getting older) Stress and performance anxiety Sexual inexperience or inadequacy Conflicting gender or sexual orientation Interpersonal factors Lack of partner Perceived unattractiveness Fastidiousness with nonsexual aspects Interpersonal conflicts Lack of desire Inadequate foreplay or poor technical skill Obsession with intercourse Rushing toward orgasm Communication problem with needs and preferences Sexual dysfunctions of the partner No time for adventure; predictable or boring sexual routine Privacy issues Sociocultural factors Inadequate sex education Antagonistic religious or family values Cultural taboos Gender discrimination

**TABLE II:** Basic biochemical investigations for women presenting with low libido^45^

General
Thyroid-stimulating hormone, iron stores
Specific
Estradiol + follicle-stimulating hormone (for diagnosis of hypothalamic amenorrhea or premature ovarian failure)
Prolactin
Sex hormone binding globulin (SHBG)
Free testosterone and bioavailable testosterone
Calculated free androgen index: Total testosterone (ng/L)/SHBG (ng/L) × 100, if SHBG is in normal range
Dehydroepiandrostenedione (DHEA-S)
Early morning cortisol if adrenal insufficiency suspected

**TABLE III:** Systemic hormonal therapies for management of hypoactive sexual desire disorder^45^

Vaginal estrogen preparations improve vaginal lubrication and reduce dyspareunia and urogenital atrophy.
Systemic estrogen or estrogen–progestogen therapy assists with vasomotor and other menopausal symptoms.
Use of estrogen with or without progestogen therapy after breast cancer is indicated only for women with moderate to severe symptoms under informed patient consent and with careful monitoring for cardiovascular, thrombotic, and breast cancer risks.
Transdermal delivery of testosterone and its derivatives for temporary increase in libido, arousal, and orgasm in postmenopausal women already treated with systemic estrogen.
Testosterone therapy exceeding 6 months is indicated only if sexual function improves.
Patients with a family history of diabetes or significant obesity should be monitored for lipid profile and fasting insulin and glucose levels while on hormonal therapies.
Tibolone may be an alternative to estrogen–androgen therapies for treating postmenopausal sexual dysfunction.

**TABLE IV:** Factors that elevate the risk of developing atrophic vaginitis^95^

Hormonal: Estrogen deficiency (menopausal or premenopausal); decreased ovarian functioning; postpartum loss of placental estrogen; increased prolactin level during lactation Illness: Immunologic abnormalities Therapies: Radiation, chemotherapy, oophorectomy Anti-estrogen medications: Tamoxifen, danazol, oxyprogesterone, leuprolide, nafarelin Lifestyle: Smoking; stopping sexual activity altogether

**TABLE V:** Differential diagnosis of atrophic vaginitis^95^

Infection
Bacterial vaginosis
Trichomoniasis
Contact dermatitis or skin reaction to
Perfumes and deodorants
Powders
Panty liners
Perineal pads
Soaps
Spermicides
Lubricants
Tight-fitting or synthetic fabric

**TABLE VI:** Society of Obstetricians and Gynaecologists of Canada clinical practice guidelines for the detection and management of vaginal atrophy^60^

Guideline	Level of evidence
1. Routine clinical assessment of postmenopausal women for symptoms and signs of vaginal atrophy.	(iii-c)
2. Regular sexual activity to maintain vaginal health.	(ii-2b)
3. Consumption of pure cranberry or lingonberry juice (rather than cranberry drink) to reduce the risk of recurrent urinary tract infections.	(i-a)
4. For the treatment of local urogenital symptoms such as vaginal itching, irritation, and dyspareunia, regular application of vaginal moisturizers is an alternative to hormone replacement therapy.	(i-a)
5. Vaginal estrogen replacement therapies for vaginal atrophy:	
Conjugated equine estrogen cream	(i-a)
Sustained-release intravaginal estradiol ring	(i-a)
Low-dose estradiol tablet	(i-a)
6. Vaginal estrogen therapy for menopausal women experiencing recurrent urinary tract infections.	(i-a)

**TABLE VII:** Estradiol levels in women on Vagifem (Novo Nordisk, Princeton, NJ, U.S.A.) and aromatase inhibitor therapy^112^

		Estradiol level on Vagifem (pmol/L)		
Patient	Concurrent ai	Baseline	2 Weeks	4 Weeks
1	Letrozole	<3.0	220	40
2	Letrozole	<3.0	232	31
3	Letrozole	=3.5	77	16
4	Anastrozole	<3.0	46	2.4^a^

a Experienced a 10-day break from Vagifem before this measurement.

## APPENDIX A: SEXUAL HISTORY QUESTIONNAIRE 41,42,43

**Table:**

Functional history What functions are affected currently? When did you start noticing a change in your interest in sex? Do you have problems with arousal or getting sufficiently lubricated? Are you able to reach orgasm? How often do you experience discomfort or pain during sex? Assessment of chronic dyspareunia (SOGC guidelines) Is vaginal entry possible at all (that is, with finger, penis, speculum, tampon)? Do you get sexually aroused at the beginning of and during intercourse? Exactly when does the pain arise? During entry of penile head? With partial penile entry? With deep penetration? With movement of the penis? During or following ejaculation? Following subsequent urination? Several minutes after attempted or successful intercourse or other vaginal stimulation attempts? Is there less or greater degree of pain at times, and any idea why? Comprehensive questions related to: Acuteness or severity of the problem (global vs local) Chronicity of the problem (primary vs secondary) Circumstances underlying the problem Medical conditions and current medications Low desire: Serious illness, depression, hypothyroidism, hyperprolactinemia, central nervous system depressants, dopamine receptor blockers, selective serotonin reuptake inhibitors (SSRIs), anti-androgens Decreased arousal: Atherosclerosis, diabetes, pelvic trauma, pelvic surgery, pelvic irradiation, multiple sclerosis and other illness associated with neurogenic impairment, SSRIs, anti-estrogens, anticholinergic medications Difficulty attaining orgasm: Same as for decreased arousal Medical conditions that might contraindicate potential therapeutic options: Thromboembolism, active liver disease, hormone-responsive cancers, severe acne (estrogen, testosterone) Eating disorders, seizures (bupropion) Patient’s level of distress, reasons for seeking help, and response to previous interventions Evaluation of couple individually and together for: Sexual communication Technical skill Sexual repertoire Sample questions for women with hypoactive sexual desire disorder (HSDD) On a scale of 0–10, how would you rate your level of desire currently and when it was the highest? When was the last time you found a change in your level of desire? Anything you think was responsible? Do you have any inhibitions or thoughts that interfere with your level of desire? Do you currently participate in sexual activities despite your altered level of desire? If so, what motivates you? Any spontaneous sexual thoughts or fantasies? Are you aroused by erotic descriptions in books or sex scenes in movies? How often do you masturbate? Do you find your partner attractive? Do you find other men or women attractive? Sample questions for women with decreased arousal: When did you notice a change in your level of arousal? What do you think is responsible for the change? Do sexual thoughts, fantasies, reading a sexy passage in a book, or seeing a sexy scene in a movie “turn you on”? Does touching your different body parts (by yourself or your partner) arouse you? If so, do you feel stimulated or titillated? Does the sensation last long enough or quickly plateau? Are there any distracting feelings or thoughts that seem to inhibit these sensations? Have you ever been abused sexually, and do still suffer from negative memories that affect your enjoyment now? Does masturbation offer you real pleasure or just momentary thrill? Are there any sexual activities that you and your partner(s) engage in for pleasure? Did you try using a vibrator or other sex toys? If so, do you find them indispensable? Are you comfortable talking with your partner(s) about the kinds of stimulation you enjoy? Do you find your partner(s) responsive when you talk about sex? Have you tried using lubricants for vaginal dryness? Which ones? Do you find them helpful? Sample questions for women with difficulty achieving orgasm Have you ever had an orgasm or heard of female genital structures, such as the clitoris? Did you know that most women require stimulation of the clitoris to become fully aroused? Do you participate in sexual activities that involve stimulation of the clitoris? Does sexual stimulation give you pleasure? If yes, can you identify inhibiting feelings or thoughts that interfere with arousal and prevent orgasm? When was the last time you noticed a change in your ability to achieve orgasm? Can you identify anyone or anything that you feel might have been responsible for the change? Can you describe exactly what you feel? But are you able to achieve orgasm at all? Or does it take longer? Did you find certain type of stimulation working better than others? Do you really get distracted during an orgasm? Do you always expect to have an orgasm when you have sex? Or are you satisfied even without achieving orgasm?
